# Postural control deficits in people with fibromyalgia: a pilot study

**DOI:** 10.1186/ar3432

**Published:** 2011-08-02

**Authors:** Kim D Jones, Laurie A King, Scott D Mist, Robert M Bennett, Fay B Horak

**Affiliations:** 1 Fibromyalgia Research Unit, Schools of Nursing & Medicine, Oregon Health & Science University, 3455 SW US Veterans Hospital Road, Portland, OR 97229, USA; 2 PT Balance Disorders Laboratory, Neurological Sciences Institute, Oregon Health & Science University, 3455 SW US Veterans Hospital Road, Portland, OR 97229, USA

## Abstract

**Introduction:**

Postural instability and falls are increasingly recognized problems in patients with fibromyalgia (FM). The purpose of this study was to determine whether FM patients, compared to age-matched healthy controls (HCs), have differences in dynamic posturography, including sensory, motor, and limits of stability. We further sought to determine whether postural instability is associated with strength, proprioception and lower-extremity myofascial trigger points (MTPs); FM symptoms and physical function; dyscognition; balance confidence; and medication use. Last, we evaluated self-reported of falls over the past six months.

**Methods:**

In this cross-sectional study, we compared middle-aged FM patients and age-matched HCs who underwent computerized dynamic posturography testing and completed the Fibromyalgia Impact Questionnaire-Revised (FIQR) and balance and fall questionnaires. All subjects underwent a neurological and musculoskeletal examination. Descriptive statistics were used to characterize the sample and explore the relationships between variables. The relationships between subjective, clinical and objective variables were evaluated by correlation and regression analyses.

**Results:**

Twenty-five FM patients and twenty-seven HCs (combined mean age ± standard deviation (SD): 48.6 ± 9.7 years) completed testing. FM patients scored statistically lower on composite sensory organization tests (primary outcome; *P *< 0.010), as well as with regard to vestibular, visual and somatosensory ratio scores on dynamic posturography. Balance confidence was significantly different between groups, with FM patients reporting less confidence than HCs (mean ± SD: 81.24 ± 19.52 vs. 98.52 ± 2.45; *P *< 0.001). Interestingly, 76% to 84% of FM patients had gastrocnemius and/or anterior tibialis MTPs. Postural stability was best predicted by dyscognition, FIQR score and body mass index. Regarding falls, 3 (11%) of 27 HCs had fallen only once during the past 6 months, whereas 18 (72%) of 25 FM patients had fallen at least once. Fifteen FM patients (60%) reported falling at least three times in the past six months.

**Conclusions:**

In this study, we report that middle-aged FM patients have consistent objective sensory deficits on dynamic posturography, despite having a normal clinical neurological examination. Further study is needed to determine prospective fall rates and the significance of lower-extremity MTPs. The development of interventions to improve balance and reduce falls in FM patients may need to combine balance training with exercise and cognitive training.

## Introduction

Fibromyalgia (FM) is a chronic disorder defined by chronic, widespread pain, including axial pain and the presence of multiple tender points on physical examination [1]. Like many chronic illnesses, the symptoms of FM extend far beyond the defining criteria. In addition to pain, most patients also have other clinical signs and symptoms, such as fatigue, disrupted sleep, impaired cognition and poor physical fitness [2]. Moreover, a recent survey of 2,596 persons with FM reported balance problems as one of the top 10 most debilitating symptoms, with a reported prevalence of 45% [3]. However, the relationship between the multiple clinical variables and postural control in FM is not known.

We recently reported that FM patients compared to matched controls had significantly impaired postural control in multiple subsystems in a standardized clinical examination using the Balance Evaluation Systems Test (BESTest) [4,5]. The FM patients also scored more poorly on balance confidence and reported six times as many falls over the past six months as healthy controls (HCs). Overall, FM severity correlated significantly with balance scores using the BESTest and self-reported balance confidence measures. It was particularly notable that FM patients were unable to maintain gait speed under cognitive distraction [5]. Although that study highlighted how common measurable balance deficits are in FM, it did not objectively quantify the physiological basis for these balance problems. Toward this end, Russek and Fulk [6] examined two subsystems, sensory organization and limits of stability, with dynamic posturography. They reported that up to 34% of FM subjects scored below the fifth percentile for population norms on sensory organization conditions. While this study provided support for the common occurrence of balance problems in FM, it did investigate the potential role that common FM clinical variables may have in dysfunctional postural control.

It is possible that balance disorders in FM may be associated with specific clinical and demographic findings, such as increasing age, obesity, reduced muscle strength and impaired cognition, sensory or motor deficits, or lower-extremity myofascial trigger points (MTPs). In support of examining lower-extremity MTPs, the gastrocnemius muscle is the agonist for control of forward postural sway and must contract up to 80% of maximum in response to large perturbations. The anterior tibialis muscle is the agonist for control of backward postural sway. It is postulated that lower-limb MTPs in these muscles may result in pain-related activity and lead to suboptimal muscular coordination. Further study of FM is needed to identify the relative contribution of neural, muscular and anthropomorphic challenges to postural stability in patients with FM and to develop specific balance interventions to remediate these impairments. Additionally, many of the medications used by people with FM are associated with side effects of postural instability. For example, opioids, tricyclics, hypnotics, benzodiazepines and cardiac medications can be associated with falls in the elderly [7]. More recent evidence suggests that side effects such as dizziness may be self-limiting with newer classes of FM medications such as anticonvulsants [8]. However, no FM study has quantified the role of medications on postural control in an effort to differentiate balance dysfunction related to the disorder versus balance dysfunction related to medications.

The purpose of the current study was to determine whether people with FM, compared to age-matched HCs, have differences on a broad array of objective balance tests (termed "dynamic posturography"), including sensory, motor and limits of stability. The relationships of balance deficits with poor strength, proprioception and lower-extremity MTPs, FM symptoms, dyscognition, balance confidence and medication use were analyzed. Last, we wanted to replicate self-reported fall data that we published in 2009 [5]. By doing so, we have sought to establish the functional relevance of the dynamic posturography results by relating it to falls.

## Materials and methods

### Subjects and protocol

This was a cross-sectional study in which FM patients and age-matched HCs (ages 30 to 59 years) were evaluated by a single examiner (KDJ) for evidence of non-FM musculoskeletal problems, neurological dysfunction, FM tender points, MTPs and strength. Subjects underwent objective dynamic posturography testing (Sensory Organization Test (SOT) and Motor Control Test (MCT), then the Limits of Stability Test (LOS)) and ankle goniometry by a single physical therapist examiner. Last, subjects completed balance-, fall- and FM-related questionnaires. Testing was done on a single day between 11:00 AM and 4:00 PM. Fatigue was monitored by the physical therapists, who employed scripted fatigue assessment questions. A convenience sample of 25 FM subjects and 27 HCs underwent testing in the physical therapy unit of an academic medical center in the Pacific Northwest. FM subjects were invited to participate if they were part of a database composed of 1,500 patients with FM overseen by KDJ and RMB and had a confirmed diagnosis of FM based on the American College of Rheumatology (ACR) 1990 criteria [1]. HCs were largely university faculty, staff and graduate students. Exclusion criteria for both groups included (1) a concomitant medical illness that, as judged by the investigator, could impair balance (for example, neurological or significant musculoskeletal disease, Ménière's disease or other inner ear disease, permanent lower-limb injury, significant psychiatric disorder), (2) unable to ambulate without an assistive device, (3) currently undergoing disability evaluation or litigation, (4) self-report of an abnormal optometric or ophthalmic examination in the past year and (5) medication changes during the past three months. The study was approved by the university's Institutional Review Board (IRB00004785), and written informed consent was obtained from all subjects.

#### Primary measure: computerized dynamic posturography

Balance control consists of several neural subsystems that may be affected by FM, which can be differentiated using a clinical balance assessment tool: the Computerized Dynamic Posturography System (NeuroCom International, Inc. Clackamas, OR, USA). The Computerized Dynamic Posturography System evaluates sensory (SOT) and motor (MCT) systems and is currently the gold standard for balance assessment [9]. Dynamic posturography has been demonstrated to be reliable and valid in assessing a variety different neurological symptoms or disorders, including dizziness, vestibular disorder, Alzheimer's disease and Parkinson's disease [10-12].

This system allows quantification of postural sway under changing sensory conditions and the latency, strength, timing and symmetry of postural responses to surface movement. It is a well-studied, sensitive and safe approach to quantify balance. It characterizes spontaneous body sway by measuring displacement of the center of pressure under the feet [13].

#### Sensory Organization Test

**Figure 1:**
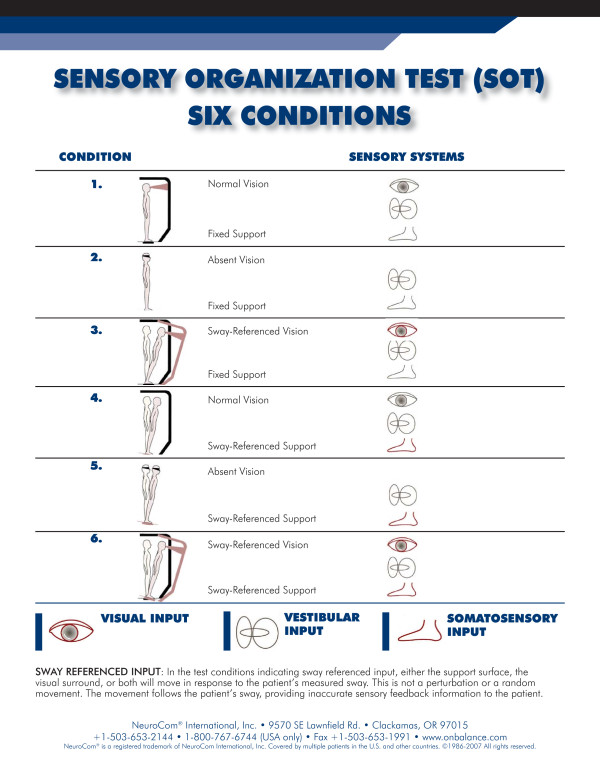
**Six conditions used to calculate Sensory Organization Test Composite scores and Visual, Vestibular, Somatosensory and Preference scores**.

The SOT protocol isolates and quantifies abnormalities in the subject's use of the three sensory systems that contribute to postural control (somatosensory, visual and vestibular). Force plates record vertical and horizontal shear forces while the patient stands under varying sensory conditions. Anteroposterior sway is compared with theoretical anteroposterior limits of stability based on the person's height and base of support. Six specific sensory conditions are tested, including eyes open with fixed platform, eyes blindfolded with fixed platform, eyes open with visual sway, eyes open with platform sway, eyes blindfolded with platform sway and eyes open with platform and visual sway. Subjects stood still with their arms at their sides and each foot on a force plate for 20 seconds while wearing a harness to prevent a fall. They were then asked to try to maintain their balance, blindfolded or with eyes open, under varying conditions. The average of three consecutive trials for each condition was calculated and used in the data summary. A score was calculated for each condition as well as a weighted average to calculate a composite score. These scores were compared to computer-generated, age-adjusted norms with a possible score of 0 to 100, with 0 being the least stable and 100 being the most stable. Additionally, the sensory protocol provides ratio scores to determine how successfully a person uses each of the sensory systems for balance. These ratio scores can help the clinician identify sensory integration deficits by manipulating the results of the six conditions (Table 1 and Figure 1). The somatosensory condition compares condition 1 with condition 2 to determine how successfully a person uses input from the somatosensory system to maintain balance. The visual ratio compares condition 4 with condition 1 to determine how successfully a person uses visual information for balance. The vestibular ratio compares condition 1 with condition 5 to determine how successfully a person uses input from the vestibular system for balance. The preference ratio determines how much a person relies on visual information, even when that information is not correct. The SOT has been tested extensively and has good test-retest reliability [14].

**Table 1:** Sensory organizational test subcomponents

Ratio	Conditions manipulated	Functional implications
Somatosensory	Condition 2/condition 1	Patient's ability to use input from the somatosensory system to maintain stability
Visual	Condition 4/condition 1	Patient's ability to use input from the visual system to maintain stability
Vestibular	Condition 5/condition1	Patient's ability to use input from the vestibular system to maintain stability
Preference	Condition 3 + 6/condition 2 + 5	The degree to which a person relies on visual information to maintain stability, even when that information is incorrect

#### Motor Control Test

This test quantifies the effectiveness of automatic postural motor responses to a horizontal force plate translation scaled to the patient's height. The response is a rapid, involuntary response to the perturbation. Measures of latency, symmetry, amplitude scaling, strength and symmetry of the reaction to backward movement were recorded. This test produces four measurements of latency, three measures of symmetry and six measures of amplitude scaling. The perturbation size ranged from small (approximately 0.5 inches over 250 milliseconds) to medium (approximately 1.25 inches over 300 milliseconds) to large (approximately 2.25 inches over 400 milliseconds). The latency times, symmetry, amplitude scaling and strength of reactions were compared between the FM and HC groups.

#### Limits of Stability Test

This test quantifies limits of stability and sway using the force plates to detect center of gravity-driven movements. Subjects stood on a force plate while focusing on a computer screen placed six feet away at eye level. They were instructed to shift their weight with knees and hips straight toward one of eight targets that were normalized to be within theoretical limits of stability. Subjects saw a line that represented their movement pattern and velocity during each eight-second attempt to move their center of gravity to the target box. The NeuroCom software then computed values for reaction time, distance, sway, velocity, end point excursion, maximal excursion and directional control.

#### Physical examination and questionnaires

The history and physical examination included a full review of body systems. Physical examinations included a focused neurological and musculoskeletal examination and measurement of height in inches and body weight in pounds (Detecto, Webb City, MO, USA). Deep tendon reflexes were measured on a scale from 0 = absent to 4+ = clonus in the right and left patellar tendons and the Achilles tendon. Plantar reflexes were rated as 0 = normal or 1 = abnormal. Foot sensation was measured with number 10 von Frey hair in three standardized plantar surfaces commonly used in diabetes research and clinical management (0 = normal or 1 = abnormal) [15]. Awareness of joint position was measured by asking the patient, with eyes closed, to identify the direction of the great toe joint position (0 = normal or 1 = abnormal). Vibratory sense was measured with a 128-Hz tuning fork bilaterally at the medial malleolus and the interphalangeal joint of the first metatarsal (0 = normal or 1 = abnormal). Cerebellar function was measured by assessing the ability to walk on the heels and toes and to move the heel up and down on opposite legs (0 = normal to 4 = too limited to test). Range of motion in both ankles was measured by goniometry in two seated positions: knee straight and knee flexed. The ankle was dorsiflexed by the physical therapist to the maximum range allowed by the patient. Range of motion in the hips bilaterally was measured using a standard clinical examination technique and was rated as 0 = normal, 1 = slightly limited, 2 = significantly limited and 3 = would not permit exam. Subjects were asked to shade in painful areas on a two-sided body diagram to determine pain locations and distribution.

The self-reported number of comorbidities which could contribute to balance deficits was measured using an investigator-designed checklist which included 15 common FM-related comorbidities (for example, irritable bowel or bladder, chronic headaches, pelvic pain) and 3 additional ailments, including diabetes and hip or knee osteoarthritis as confirmed by X-rays. The type and number of medications and the number of tablets consumed per day, both prescription and over-the-counter, were recorded.

Lower-body strength was measured using the 30-second chair stand [16]. Participants were seated in a 17-inch wooden straight-backed chair. They were asked to rise to full height with their arms crossed over their chest as many times as possible within 30 seconds. The number of stands was recorded. Higher scores indicated greater strength.

Pain was measured in three ways: a single numeric rating scale for pain (0 to 10) on the Fibromyalgia Impact Questionnaire-Revised (FIQR), the number of body regions on a two-sided body diagram where subjects reported pain (0 to 24) and total myalgia score. The total myalgia score was summed by eliciting pain on 18 tender points as described in the ACR 1990 criteria for FM [1]. Pain was rated as 0 (no pain) to 3 (withdrawal from the examiner). FM patient scores ranged from 11 to 54, with higher scores indicating more pain. Two bilateral MTPs critical to normal ambulation were assessed in this study according to the Trigger Point Manual criteria [17]: gastrocnemius insertion of the Achilles tendon and the proximal insertion of the anterior tibialis (0 = absent or 1 = present).

Falls were evaluated by asking participants to report the number of falls they had sustained in the past 6 months. Falls were defined as unintentionally coming to rest at a level at or below the floor (that is, tripping alone did not count as a fall). Falls related to participation in sporting activities were not included.

Balance confidence was measured using the Activity-Specific Balance Confidence Questionnaire (ABC). The ABC is a valid and reliable questionnaire on balance confidence in accomplishing 16 common mobility tasks, such as walking around the house, riding an escalator with or without holding a rail, being bumped while walking in a crowded mall and standing on a chair. Confidence is measured from 0% to 100%, with higher scores indicating greater confidence in keeping one's balance [18]. The ABC is a valid predictor of falls in elderly patients. These researchers further demonstrated that scores < 50 are indicative of persons in home care with low functional capacity and that scores between 50 and 80 correlate with moderate functional levels (for example, ambulatory nursing home residents) [19]. Cronbach's α was 0.983 with an interitem correlation of 0.785.

FM-specific physical function and symptom severity was measured using the FIQR. This is a 21-item questionnaire using a 0 to 10 scale that measures physical functioning, overall well-being, depression, anxiety, sleep, pain, stiffness, fatigue, tenderness, balance and environmental sensitivity, as well as well-being over a seven-day period in subjects with FM. HCs completed the Symptom Impact Questionnaire, which is the same as the FIQR but replaces the word "fibromyalgia" with the word "health" [20]. The FIQR has been shown to have construct validity, good test-retest reliability, content relevance and sensitivity to change for improvement and decline of health status [21]. Total FIQR scores range from 0 to 100, with higher scores indicating a greater negative impact of FM. The Cronbach's α was 0.976 with an interitem correlation of 0.657.

Cognitive functioning was measured using the Multiple Ability Self-Report Questionnaire (MASQ). The MASQ is a 38-item self-report measure assessing five domains of self-perceived cognitive function: language, visual-perceptual ability, verbal memory, visual spatial memory and attention/concentration. The content validity of MASQ items and domain groups was established by expert rating of neuropsychologists and demonstrated concurrent validity with objective neuropsychological measures in multiple populations. Scores range from 38 to 190, with higher scores indicating poorer cognitive function [22]. Use of the MASQ has been reported in a FM trial with scores significantly lower than HCs and improvement with milnacipran [23]. National Institutes of Health-funded FM trials of the MASQ produced similar data and are under review for publication [24]. The Cronbach's α was 0.963 with an interitem correlation of 0.410.

### Statistical analysis

These analyses are exploratory and designed to estimate the effect size of relationships between FM status and balance. We used descriptive statistics to characterize the sample and explore the relationships between falls, balance and selected secondary outcomes. The primary objective was to examine differences in the retrospective fall prevalence and balance perturbation of FM subjects compared to the age-matched HCs. As the distribution of the data was non-normal, we conducted nonparametric two-sample Kolmogorov-Smirnov tests to compare differences. We corrected for multiple comparisons using the Holm-Bonferroni adjustment [25,26]. We used correlations to investigate relationships between subjective, clinical and objective variables, including retrospective fall prevalence, balance perturbation to strength, range of motion, total myalgia score, ABC, FIQR, MASQ and selected clinical and demographic variables, including medications. Finally, among the FM patients, an *a priori *model of balance (SOT composite score) was tested on the basis of FIQR, medications (class and total number of daily tablets), cognition (MASQ), strength and body mass index (BMI).

### Demographics and clinical variables

The total sample was generally middle-aged (mean age ± SD: 48.6 ± 9.7 years), primarily female (88.4%), Caucasian (90.4%), non-Hispanic (94.2%) and in a long-term relationship (75% married or with a long-term partner). There were no differences between the groups on these items; however, the groups differed with regard to education and income. Specifically, 66% of HCs had completed some postgraduate education, but only 20% of FM subjects had graduated from college. Fifty-five percent of the HCs' annual household income was > $100,000 compared with 44% of FM patients living in households with < $50,000 annual income (Table 2).

**Table 2:** Demographic differences between groups^a^

Variable	Healthy controls	Fibromyalgia	Statistical significance
Mean age, years (± SD)	46.5 (± 10.9)	50.8 (± 7.7)	NS
Females	89%	88%	NS
Caucasian	92.6%	88.0%	NS
Married	77.8%	72.0%	NS
Highest education level			0.002
High school	7.4%	8.0%	
Trade school or some college	0.0%	8.0%	
College graduate	25.9%	64.0%	
Postgraduate education	66.7%	2.0%	
Annual household income			0.045
< $30,000	7.6%	24.0%	
$30,000 to $70,000	26.9%	36%	
$70, 000 to $100,000	7.6%	24.0%	
> $100,000	57.7%	24.0%	

^a^SD: standard deviation.

## Results

### Falls, sensory organizational and balance confidence

FM patients reported significantly more falls over the past six months than HCs. Specifically, 3 (11%) of 27 HCs had fallen only once during the past six months, whereas 18 (72%) of 25 FM patients had fallen at least once, with 15 FM patients (60%) having fallen more than three times in the past six months.

**Figure 2:**
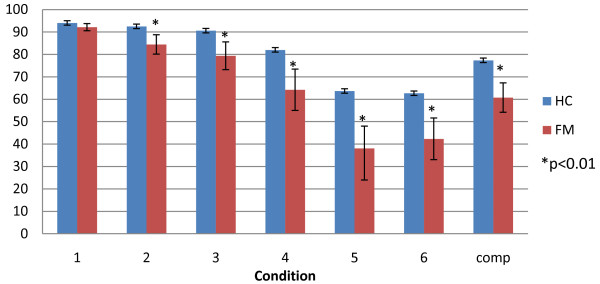
**Mean values and standard deviations between patients with fibromyalgia (FM) and healthy controls (HC) on six conditions and Sensory Organization Test Composite scores**.

The SOT composite balance scores were significantly different between groups (Figure 2). All conditions except quiet stance with eyes open (condition 1) differed. The ratio scores, vestibular visual and somatosensory, were significantly impaired in FM patients compared to HCs. However, there was no difference between groups with regard to preference for any one sensory system over another to maintain postural stability.

Balance confidence on the ABC was also significantly different between groups, with FM patients reporting less confidence scores (mean ± SD: 81.24 ± 19.52 vs. 98.52 ± 2.45; *P *< 0.001). Additionally, three FM subjects (6%) scored < 50, six FM subjects (12%) scored between 50 and 80 and sixteen FM subjects (64%) scored > 80 (Table 3).

**Table 3:** Falls, sensory organizational test ratio scores and balance confidence between groups^a^

Measured variable	Healthy controls	Fibromyalgia	Statistical significance
Reported falls	0.15 (± 0.36)	3.48 (± 3.64)	0.008
SOT Comprehensive	77.33 (± 7.72)	60.72 (± 15.9)	0.010
Vestibular	0.67 (± 0.16)	0.41 (± 0.26)	0.013
Visual	0.87 (± 0.76)	0.69 (± 0.23)	0.017
Somatosensory	0.98 (± 0.34)	0.91 (± 0.10)	0.025
Preference	0.98 (± 0.10)	100 (± 25)	NS
ABC	98.52 (± 2.46)	81.24 (± 19.53)	0.000

^a^SOT: Sensory Organization Test; ABC: Activity-Specific Balance Confidence Questionnaire. All data are means (± SD).

### Prediction of sensory organization for balance

Using an *a priori *model, regression analyses demonstrated that four variables predicted the primary balance measure (SOT composite score): visual spatial memory (*P *= 0.005), verbal memory (*P *= 0.000), FIQR score (*P *< 0.001) and BMI (*P *= 0.036). Type or number of medications, pain and strength, though different between groups, did not predict balance control. When controlling for education and income (*P *= 0.099), all of the predictors remained significant within the model and the overall explained variance of the model had only a minor improvement (Δ*R*^2 ^= 0.0251; *P *= 0.267) (see Additional file 1).

### Motor control tests and limits of stability

The only measure in response to perturbation that differed between the two groups was the latency of response to medium backward perturbation. Medium backward latencies were slower in FM patients compared to HCs (260 ± 16.1 milliseconds vs. 245 ± 17.6 milliseconds, respectively; *P *< 0.001), with no differences noted in small or large backward perturbations. No differences were found between groups in amplitude scaling or weight symmetry on motor control tests. Responses to forward perturbations were similar between groups. When patients were tested for voluntary movements on limits of stability, they exhibited no difference in reaction time, velocity, maximal excursion or directional control. The only difference noted was that FM patients had a slightly shorter end point excursion than HCs (mean ± SD: 73 ± 8.3 cm vs. 78 ± 10.3 cm; *P *= 0.047).

### Comorbidities

The average FM subject had had FM for > 10 years, but only 48% had been formally diagnosed for > 10 years. The clinical characteristics between groups differed, as expected, with FM subjects reporting more comorbidities, less functional strength, higher BMI and more pain and tender points than HCs. Specifically, gastrocnemius and anterior tibialis MTPs were found in 76% to 84% of FM subjects and in 4% to 8% of HCs (Table 4). The most common comorbidities reported in the FM group were restless leg syndrome (76.0%), irritable bowel syndrome (72.0%) and chronic headache (68.0%). In exploring FIQR line item questions, we found that the symptoms rated as most severe were poor sleep and fatigue and those rated as least severe included anxiety and depression. Self-reported balance on the FIQR correlated to SOT Comprehensive score at *r *= -0.61 (*P *= 0.0000). There were no differences between groups with regard to neurologic examinations or range of motion of the hip, knee and ankle (goniometry).

**Table 4:** Clinical differences between groups^a^

Variable	Healthy controls	Fibromyalgia	Statistical significance
Mean number of concurrent medical problems (*N *= 17) (± SD)	1 (± 1.2)	8 (± 3.6)	0.000
Mean chair stands in 30 seconds (± SD)	19.4 (± 4.9)	13.0 (± 10.5)	0.000
Body mass index (± SD)	26.3 (± 5.6)	30.7 (± 5.8)	0.004
Anterior tibialis MTP present, %	3.7%	84.0%	0.000
Gastrocnemius MTP present, %	7.6%	76.0%	0.000
Mean number of pain areas marked on body mannequin (± SD)	1.35 (± 2.46)	16.40 (± 6.33)	0.000
Mean number of ACR tender points (± SD)	1.56 (± 1.95)	16.04 (± 2.11)	0.000
Mean total myalgia score (± SD)	1.78 (± 2.42)	35.32 (± 10.10)	0.000
Mean total FIQR score (± SD)	5.00 (± 4.58)	54.06 (± 17.75)	0.000
Mean pain score on FIQR (± SD)	0.67 (± 0.83)	5.88 (± 2.18)	0.000
Mean imbalance score on FIQR (± SD)	0.22 (± 0.50)	4.04 (± 2.87)	0.000

^a^MTP: myofascial trigger point; ACR: American College of Rheumatology; FIQR: Fibromyalgia Impact Questionnaire-Revised.

### Medications

An aggregate total of 48 classes of medications were taken by HCs and FM subjects. The average FM subject took six classes of medication and consumed eight tablets daily compared to 1.8 classes consumed as 2.4 tablets among the HCs (*P *< 0.001). FM subjects took central nervous system (CNS)-acting medications thought to affect balance, including sedative hypnotics (*n *= 2), benzodiazepines (*n *= 1), tricyclics (*n *= 8) and antihypertensives (*n *= 8). Eight of twenty-five FM subjects were taking opioids. Taking opioids did not correlate with BMI, total FIQR, chair stands, self-reported falls or SOT Comprehensive scores.

### Cognition

FM subjects scored worse than HCs on all five cognition measures in the MASQ (Table 5).

**Table 5:** Cognition differences between healthy controls and patients with fibromyalgia^a^

MASQ sections	Healthy controls	Fibromyalgia	Statistical significance
MASQ: Language subscale	12.56 (± 3.61)	19.80 (± 4.81)	0.000
MASQ: Visual Perceptual Ability	10.56 (± 3.07)	14.36 (± 4.37)	0.001
MASQ: Verbal Memory	14.44 (± 4.07)	21.92 (± 3.89)	0.000
MASQ: Visual Spatial Memory	12.67 (± 3.13)	18.80 (± 3.76)	0.000
MASQ: Attention Concentration	14.63 (± 3.56)	21.32 (± 4.72)	0.000

^a^MASQ: Multiple Ability Self-Report Questionnaire. All data are means (± SD).

### Tolerability and adverse events

No HCs responded to scripted fatigue questions in a manner that suggested they needed to rest. Conversely, five FM subjects did rest during some part of dynamic posturography testing. Resting included being unharnessed from the NeuroCom and sitting in a chair for three to five minutes. There were no unharnessed falls or adverse events during this protocol.

## Discussion

Balance or postural stability is a complex task that involves the rapid and dynamic integration of multiple sensory, motor and cognitive inputs to execute appropriate neuromuscular activity needed to maintain balance [27]. In recent surveys, persons with FM reported balance problems as one of the top 10 most debilitating symptoms, with a reported prevalence of 45% to 68% [28]. On the basis of mounting self-report data on postural instability, we have attempted to objectively measure why postural stability could be problematic in FM. We propose that FM likely affects dynamic balance control because of altered somatosensory inputs to the CNS. Somatosensory input from muscle spindles, Golgi tendon organs and superficial and deep cutaneous afferents are the primary sensory inputs used for postural orientation in space and for automatic postural response [27,29,30]. FM is defined by abnormal perception of pain with light somatosensory stimulation. It is possible that multiple pain processing dysfunctions in FM may lead to poor balance. Moreover, fall prevention requires rapid and often multiple automatic corrections coordinated by the CNS. There is evidence about multiple processing abnormalities in the CNS, including cognitive dysfunction being linked to postural instability [27].

This study produced some novel findings. Compared to HCs, FM subjects have (1) consistent sensory deficits on dynamic posturography despite a normal clinical neurological examination; (2) poorer scores on all balance- and FM-related questionnaires, less strength, more pain areas and higher total myalgia scores but not anxiety and depression scores; (3) gastrocnemius and anterior tibialis MTPs were found in 76% to 84% of FM subjects, leading to the conjecture that active MTPs in these leg muscles may affect balance and falls as patients attempt to maintain postural stability through activation of the anterior and posterior leg muscles [31]; (4) postural stability is best predicted by FM severity (FIQR), cognitive impairment (MASQ) and BMI; the use of opioids and/or benzodiazepines, the total number medication tablets consumed per day, and muscle strength or pain scores did not predict objective measures of balance; (5) motor tests, including limits of stability, were largely normal with the exception of longer latencies to backward perturbations and shorter end point excursion on limits of stability; and (6) these data confirm our earlier report of significantly more self-reported falls in FM patients compared to HCs.

To the best of our knowledge, computerized dynamic posturography in FM has been reported only once before. Russek and Fulk [6] tested 32 female FM patients (mean age ± SD: 52 ± 14 years) with the same NeuroCom sensory organization test with limits of stability. Their SOT data are consistent with the results of our study. We found slightly lower SOT Comprehensive scores (60 vs. 65) with additional impairments in the Visual and Vestibular sections. Although we found a significant difference in somatosensory scores between groups (0.94 vs. 0.91), Russek and Fulk did not. However, the absolute values derived from both FM studies clearly indicate more impairment in visual and vestibular control. Bayazit *et al*. [32] suggested that women with FM have neural brainstem disintegration based on an abnormal auditory brainstem response. This notion supports both the current study results and Russek and Fulk's findings of vestibular impairments. Another area of agreement is the finding that the less taxing sensory component tasks were normal, with deficits worsening as test condition complexity increased.

No evidence of malingering was displayed in the objective data patterns obtained during posturography in the FM subjects. Other groups have identified malingerers as those who have substandard scores in condition 1 or exhibit large intertrial variation, particularly in conditions 1 and 2 compared to conditions 5 and 6 [33,34]. Malingering is also more likely when sway is less with eyes open in a sway-referenced visual surrounding (conditions 3 and 6) than with eyes closed (conditions 2 and 5, respectively). These data may inform those who continue to describe FM as a functional somatic syndrome and doubt the veracity of FM patients' complaints [35].

Interestingly, the balance confidence scores in Russek and Fulk's study [6] were slightly lower than those in our sample (70.1 vs. 81.2). The percentage of subjects scoring extremely low for their age was also greater in the Russek and Fulk study. Therefore, they reported worse overall balance confidence as well as a higher percentage score in the group consistent with nursing home patients or elderly patients receiving home care. Nonetheless, in both studies, balance confidence, as measured by the ABC questionnaire, correlated with SOT composite scores, suggesting that people with FM are well aware of their objective balance deficits. In both studies, SOT composite scores and ABC scores were correlated with disease severity (FIQ or FIQR), perhaps indicating that FM symptom severity and poor physical function are also related to postural control and balance confidence. Nonetheless, the differences in the objective (dynamic posturography) and subjective (ABC questionnaire) balance scores are not fully explained by the data. Perhaps Russek and Fulk's subjects were more distressed, since they were recruited from a support group and our subjects were recruited from a rheumatology practice. Alternatively, the difference may be due to random variation, self-selection or small sample size. Another major difference in the two studies is that we studied subjects' medical histories, physical examinations, cognition and medications, which required us to have a control group. Russek and Fulk used well-validated but computer-generated, age-matched norms for comparison, thus limiting their ability to explore the relationship between critical FM clinical variables and postural control.

The relevance of the frequent occurrence of gastrocnemius and anterior tibialis MTPs in the FM subjects is essentially conjectural at this time. More detailed analyses of lower-body MTPs in the current study was confounded by group. It may be relevant that Bazzichi *et al*. [36] reported surface electromyography (EMG) responses in 100 women with FM and 50 HCs while evaluating the anterior tibialis and distal part of the vastus medialis during isometric contraction. They found that FM patients had a significantly impaired fatigue index and a decrement of normalized median electrical frequency in the anterior tibialis and distal part of the vastus medialis during isometric contraction. Similarly, Leveille *et al*. [37] prospectively followed > 700 community-dwelling adults > 70 years old and found that chronic musculoskeletal pain predicted falls and poor balance. In a follow-up regarding the same cohort, Eggermont *et al*. [38] recently reported that mobility problems are highly predicted by tender point counts. Further study is needed to determine whether pain at MTPs, in muscles needed to lift and then push off the forefoot during ambulation, could aggravate the risk for falls in persons with FM.

Despite minimal information about consistent areas of balance deficits, some researchers have found improvements in Romberg's test (eyes opened, closed or ratio) or one-legged stance in response to multimodal exercise programs with a significant strength training element for patients with FM [39-41]. Trials of magnetic pulse therapy or vibratory exercise have also demonstrated balance improvements in FM patients, although the mechanisms of action are not well understood [42,43]. Most recently, clinical trial data indicated one-legged stance improvement with Tai Chi and yoga exercises with mindfulness modified for FM [44,45]. These interventions, although not specific to the type of balance deficits we report here, are critical because poor balance and fear of falling with six other variables best predicted lower functional ability in a regression model used to study 1,735 women with FM [46].

With regard to falls, there are no interventional data to reduce falls in FM patients. However, Rutledge *et al*. [47] have recently taken a key step toward that goal by reporting the first prospective falls data in FM patients. Their six-month descriptive longitudinal study employed standardized fall diaries with all reported falls followed up by telephone administration of the Fall Interview Guide. Eight-eight women with FM (median age 57 years, age range 21 to 69 years) reported 37 falls and 193 near-falls during the study. Interview data indicated that intrinsic factors, such as dizziness or feeling off-balance, were associated with almost all falls or near-falls. Extrinsic factors such as uneven surfaces, wet or slippery surfaces and objects in the pathway were less commonly associated with falls or near-falls. These data support earlier retrospective reports indicating that falls are common in people with FM. Further investigation is needed to identify other intrinsic fall risk factors that may be unique to FM patients, as interventions to reduce falls in the elderly in general may not be sufficient to prevent falls in this patient population.

The current study may further inform therapeutic goals for a fall prevention program in FM. Because integration of information from all sensory systems appears to be impaired in this population, it is important for clinicians such as physical therapists and exercise instructors to include sensory challenges in their exercise sessions. For example, practicing a task without the use of vision, practicing standing tasks on foam or walking while slightly turning the head encourages patients to adapt their reliance on sensory inputs for balance. Similarly, because cognition was found to correlate strongly with balance control in this population, practicing dual cognitive tasks while walking and balancing may be of value. On the basis of our findings, the clinician could also consider the number of comorbidities, cognitive training and careful monitoring of the effects of medication. Bearing in mind the possible relevance of active lower-limb MTPs to postural imbalance in some patients, future researchers could assess the efficacy of specific MTP therapies [2]. Another element of possible importance in balance therapy for FM patients is how one reacts to being pushed off balance. We found that FM subjects scored lower on the midrange perturbations. When the postural perturbation was in the midrange, the FM subjects had difficulty recovering their balance, so balance reactions such as these should be incorporated into physical therapy or exercise rehabilitation in this patient population.

A limitation in fully explaining the findings regarding balance and falls in the current study is that gait was not evaluated. However, other researchers have reported gait abnormalities in FM patients. Heredia Jiménez *et al*. [48] reported that 55 women with FM compared to 44 controls exhibited significant differences in gait velocity, stride length, cadence, single- and double-support ratio, stance and swing phase ratio using the GAITRite System (CIR Systems, Inc., Havertown, PA, USA). Furthermore, each of these decrements correlated with the total FIQ score, such that persons with greater impairment from FM had poorer gait performance. Similarly, Auvinet *et al*. [49] used computerized gait analysis of 14 middle-aged women with FM and 14 matched controls and found similarly altered results in stable walking. Also, Pierrynowski *et al*. [50] reported that women with FM walk with different muscle recruitment patterns compared to controls. It is not known whether these patterns develop to minimize pain during ambulation. Indeed, Graven-Neilsen *et al*. [51] suggested that increased activity of antagonistic muscle and decreased activity of agonistic muscle are consistent with Lund *et al*.'s [52] pain adaptation model. Graven-Neilsen *et al*.'s hypothesis was based on a study in which hypertonic saline was infused into either the anterior tibialis or gastrocnemius muscle and monitored by EMG during treadmill walking [53]. Taken as a whole, these studies suggest the need to further explore the relationship between lower-extremity MTPs in FM patients and postural stability and falls.

A novel finding in this study was the strong link between balance and cognition. For the past decade, researchers have amassed substantial cross-sectional data documenting cognitive dysfunction in FM patients, first suggesting that FM patients performed similarly to HCs who were 20 years older [54]. Multiple studies in both FM and widespread pain reveal self-reports of concentration, alertness and memory problems as well as challenges in completing demanding cognitive tasks [55-58]. Cross-sectional data are emerging that indicate that physical performance predicts attention and speed of cognitive processing in FM [58]. The relationship between poor fitness and impaired cognition in FM patients is plausible and requires further investigation [59-62].

### Limitations

As this was a pilot study, the sample size was small (*n *= 25 FM patients vs. 27 HCs). As it was exploratory, it had multiple dependent variables. However, the likelihood of reporting spurious findings was minimized by using the Bonferroni correction and setting the *P *value at < 0.01. In fact, most *P *values were < 0.001. One area where sample size may have been a factor was in our regression model regarding medications in FM patients. As few patients (*n *= 8 of 25) were taking opioids, hypnotics or benzodiazepines, a larger study is needed to confirm the lack of association between balance and CNS-acting medications in FM patients. Alternatively, FM subjects could be tested while on and off CNS-acting medications to clarify the role of these agents in postural stability. However, in a three-year prospective study of 1,002 community-dwelling women ages 65 years and older with chronic widespread pain, an alarming percentage of women fell each subsequent year (39%, 36% and 39%, respectively). These researchers reported a protective effect of analgesic medication use in reducing fall rates and concluded that preventing falls in patients with widespread pain requires a multifactorial treatment approach that includes the use of pain medications [63].

Another possible limitation of our study is that we relied on self-reports of normal vision on the basis of a visit to an optometrist or ophthalmologist in the past year. Future researchers could measure FM patients' visual acuity directly. As expected, the HCs had more education and higher incomes. The HCs were largely academic employees. These variables were not generally associated with postural stability. Moreover, when adjusting for these differences, significance levels did not change. The examiners were not blinded to group, but this potential limitation was minimized because of the objective nature of computerized posturography and the use of self-reports rather than examiner-administered questionnaires.

Another limitation of the study was the retrospective nature of fall reporting and the lack of inquiries regarding the consequences of falls (for example, fracture, further limitation in physical activity due to fear of falling). Moreover, we did not question subjects regarding intrinsic versus extrinsic variables that may have contributed to each fall. For example, falls related to attempts to rapidly get to a bathroom because of irritable bowel, bladder and/or stress urinary incontinence is plausible in this population. The finding that 15 of 25 subjects fell three or more times indicates that falls were commonly experienced by most FM subjects and that scores were likely not led by outliers. It is unlikely that adjustment to new medication was related to falls in this study, as subjects were required to be on a stable dose of medications for at least the past three months. It is also unlikely that advancing age was related to falls, as most of our sample was middle-aged (mean age 50.8 years), with the oldest participant being 59 years of age. We purposely recruited a younger sample to minimize falls and balance perturbations related to age. Twice we have demonstrated a significantly greater number of falls in FM patients compared to age-matched controls. In another study of falls in 70 community-dwelling people with FM ages 50 years and older, Jones *et al*. [46] found the following variables best predicted falls: gait velocity, cognitive performance, number of cardiovascular drugs, total number of drugs, age, uncorrected vision, perceived postural instability, clinical balance testing and lower-body strength. However, only 42 of 70 persons self-reported falls in the past year, which was considerably lower than the data reported in the current study or by Jones *et al *[5]. Jones *et al *[46] did not quantify FM severity in their study, and thus it is not known whether FM severity was a potential predictor of falls. Prospective validation of self-reported falls and their negative consequences is needed.

Another area for further study of postural stability in FM patients is the role of BMI. BMI was higher in FM compared to HCs, and this difference remained in a regression model that predicted sensory balance deficits. However, the literature is mixed in implicating overweight versus morbid obesity with regard to falls or postural stability [64-68]. It is not known whether higher body weight impairs balance control. It is likely that muscle strength does not increase in proportion to total weight in obese individuals; therefore, obese persons may not be able to generate adequate force required to quickly regain postural control. This study was not designed to determine whether obesity was confounded by poor physical fitness. We did find, however, that BMI did not correlate with scores on the FIQ-R Physical subscale (*r *= -0.0304, *P *= 0.8852). Future studies could explore this potential relationship by determining aerobic capacity or other standardized laboratory measures of fitness.

The computerized dynamic posturography results of the middle-aged adults with FM in this study yielded scores that are comparable to those of healthy persons in their eighth decade of life, based on normative computer-generated data. Thus, the balance deficits and fall frequency reported here are clinically significant. The main targets of physical and rehabilitative medicine at this point are the relief of symptoms and an improvement in the activities of daily living for patients with FM. Balance training is generally not included in FM treatment, because it is not known what aspect of balance, if any, is involved in FM. We believe that balance impairment is an important component of FM and is currently not being assessed in the clinic or being treated by therapists. Balance impairment can negatively affect patients' quality of life by increasing the risk of falling, fall-related injuries and fear of falling [69]. By identifying and quantifying consistent balance abnormalities in FM patients, we hope to improve the effectiveness and specificity of treatments as well as to more effectively assess outcomes of treatments in relieving balance difficulty. More practically, balance training and fall prevention as an aspect of rehabilitation are not currently the standard of care.

## Conclusions

This study shows that middle-aged people with FM have consistent objective sensory deficits as measured by dynamic posturography despite a normal clinical neurological examination. FM patients also scored more poorly on all balance and FM-related questionnaires, strength, number of pain areas and total myalgia score. Interestingly, 76% to 84% of FM patients had gastrocnemius and/or anterior tibialis MTPs. Postural stability was best predicted by FM severity as determined by the FIQR, cognitive impairment as determined by the MASQ, and BMI. On the other hand, the use of opioids and benzodiazepines, the total number medication tablets consumed per day, muscle strength and pain scores did not predict balance problems. These data confirm our earlier report of significantly more self-reports of falls by FM patients compared to HCs. Further study is needed to determine whether balance, cognitive and exercise training can improve postural stability and reduce falls in this population.

## Abbreviations

ABC: Activity-Specific Balance Confidence Questionnaire; ACR: American College of Rheumatology; BESTest: Balance Evaluation Systems Test; BMI: body mass index; CNS: central nervous system; EMG: electromyography; FIQR: Fibromyalgia Impact Questionnaire-Revised; FM: fibromyalgia; HC: healthy control; LOS: Limits of Stability Test; MASQ: Multiple Ability Self-Report Questionnaire; MCT: Motor Control Test; MTP: myofascial trigger point; SOT: Sensory Organization Test.

## Competing interests

The authors declare that they have no competing interests.

## Authors' contributions

KDJ participated in all phases of the study, including study design; oversight of MS students; data collection, entry, analyses and interpretation; and final manuscript preparation. LAK participated in balance-related contributions to study design, analyses, interpretation and final manuscript preparation. SDM performed all statistical analyses and final manuscript preparation. RMB participated in all phases of the study, including study design, analyses, interpretation and final manuscript preparation and provided funding from the Fibromyalgia Information Foundation. FBH participated in balance-related contributions to study design, analyses, interpretation and final manuscript preparation and provided access to the Clinical Research Dynamic Posturography System (NeuroCom International, Inc.) and National Institute on Aging funding to support the physical therapist who collected posturography data. All authors read and approved the final manuscript.

## Supplementary Material

Additional file 1 **Additional file **1**contains data output to support the reported regression model parameter estimates of independent variables in relation to the Sensory Organization Test Composite score for patients with fibromyalgia**. Click here for file
