# Supplementary material for: Postural control deficits in people with fibromyalgia: a pilot study
Source: Arthritis Res Ther. 2011 Aug 2;13(4):R127. doi: 10.1186/ar3432 (PMC3239367; doi:10.1186/ar3432)
Supplement: Additional file 1 — contains data output to support the reported regression model parameter estimates of independent variables in relation to the Sensory Organization Test Composite score for patients with fibromyalgia. [file ar3432-S1.DOCX]

Table for Additional File 1. Regression model parameter estimates of

independent variables in relation to the SOT composite score for fibromyalgia subjects

| Overall Significance  p<0.000 | | | R^2^ = 0.567 | Adjusted R^2^ = 0.532 | |
| --- | --- | --- | --- | --- | --- |
| Variable | Coefficient | Standard Error | Significance | 95% Confidence Interval | |
| Visual Spatial | 1.47 | 0.49 | 0.005 | 0.48 | 2.47 |
| Verbal | -1.29 | 0.46 | 0.008 | -2.21 | -0.36 |
| FIQ-R | -0.37 | 0.08 | 0.000 | -0.53 | -0.21 |
| BMI | 0.55 | 0.25 | 0.036 | 0.04 | 1.07 |
| Constant | 64.42 | 9.47 | 0.000 | 45.37 | 83.47 |

FIQR: Fibromyalgia Impact Questionnaire-Revised; BMI: body mass index; SOT: Sensory Organization Test

Additional file 1 contains data output to support the reported regression model parameter estimates of independent variables in relation to the SOT composite score for fibromyalgia subjects
